# Echocardiography-guided tricuspid valve replacement by extracted extracellular matrix in post-traumatic tricuspid regurgitation

**DOI:** 10.1007/s10554-020-01965-8

**Published:** 2020-08-14

**Authors:** Paweł Czub, Monika Budnik, Adam Arendarczyk, Janusz Kochanowski

**Affiliations:** Medical University of Warsaw, Warsaw, Poland

Extracted extracellular matrix (Cor Matrix) applies growth factors, matricellular proteins, glycosaminoglycans and adhesion factors. After implantation into human tissue, CorMatrix functions as a scaffold onto which the body’s own cells migrate. The matrix itself dissolves after 6–8 months, leaving new leaflets built of patient fibroblasts. Until now it was used mainly as a patch in intracardiac shunts and in patients with endocarditis.

**Fig. 1:**
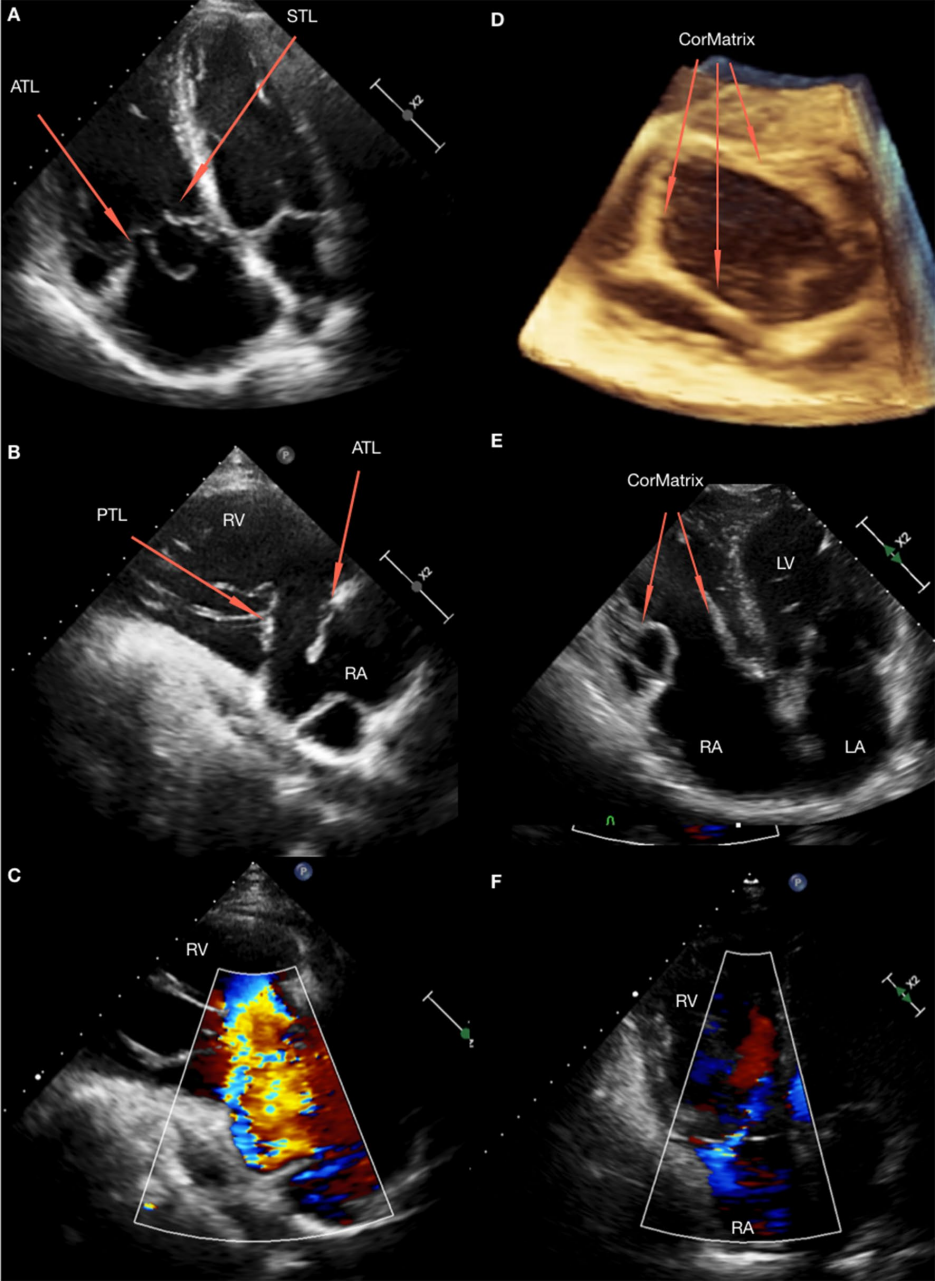
**a**, **b** Transthoracic echocardiography at admission- tricuspid valve leaflets, prolapse of the anterior leaflet, RV enlargement. **c** Transthoracic echocardiography at admission- severe tricuspid regurgitation. **d** 3D Intraoperative transesophageal echocardiography—tricuspid reconstruction using CorMatrix. **e** Follow-up transthoracic echocardiography—reduction of RV enlargement, proper valve function. **f** Follow-up transthoracic echocardiography—mild tricuspid regurgitation. *ATL* anterior tricuspid leaflet, *LA* left atrium, *LV* left ventricle, *PTL* posterior tricuspid leaflet, *RA* right atrium, *RV* right ventricle, *STL* septal tricuspid leaflet

A 49-year old patient with severe tricuspid regurgitation secondary to prolapse of the anterior leaflet as a result of the rupture of the chordae tendineae following non-penetrating chest trauma with features of progressive symptomatic right ventricular (RV) dysfunction was admitted to the Department of Cardiac Surgery. In echocardiography significant RV and right atrium dilatation was diagnosed (RVIT AP4C basal 7.5 cm, RAA 46 cm^2^, RAV 236 ml) as well as tricuspid annulus dilatation to 58 mm (Fig. 1a–c). The tricuspid valve was reconstructed using CorMatrix material. The valve was sewn before chest opening to fit with the size of the ring and the distance of the leaflets to the papillary muscles. The loci of attachment for papillary muscles have now been reconstructed. Intraoperatively, good coaptation of the leaflets with only mild regurgitation was observed (Fig. 1d). Postoperative ECHO revealed normal valvular function, with mild regurgitation without increase of transvalvular gradient. The results of controlling echocardiography performed with intervals of 6 months, a year, two and three years, revealed a good treatment effect and proper valve function. Moreover, RV dimension has significantly decreased (Fig. 1e, f).

